# Genomic Ancestry of North Africans Supports Back-to-Africa Migrations

**DOI:** 10.1371/journal.pgen.1002397

**Published:** 2012-01-12

**Authors:** Brenna M. Henn, Laura R. Botigué, Simon Gravel, Wei Wang, Abra Brisbin, Jake K. Byrnes, Karima Fadhlaoui-Zid, Pierre A. Zalloua, Andres Moreno-Estrada, Jaume Bertranpetit, Carlos D. Bustamante, David Comas

**Affiliations:** 1 Department of Genetics, Stanford University, Stanford, California, United States of America; 2 Institute of Evolutionary Biology (CSIC-UPF), Universitat Pompeu Fabra, Barcelona, Spain; 3 Department of Biological Statistics and Computational Biology, Cornell University, Ithaca, New York, United States of America; 4 Laboratory of Genetics, Immunology, and Human Pathology, University Tunis El Manar, Tunis, Tunisia; 5 The Lebanese American University, Chouran, Beirut, Lebanon; University of Aarhus, Denmark

## Abstract

North African populations are distinct from sub-Saharan Africans based on cultural, linguistic, and phenotypic attributes; however, the time and the extent of genetic divergence between populations north and south of the Sahara remain poorly understood. Here, we interrogate the multilayered history of North Africa by characterizing the effect of hypothesized migrations from the Near East, Europe, and sub-Saharan Africa on current genetic diversity. We present dense, genome-wide SNP genotyping array data (730,000 sites) from seven North African populations, spanning from Egypt to Morocco, and one Spanish population. We identify a gradient of likely autochthonous Maghrebi ancestry that increases from east to west across northern Africa; this ancestry is likely derived from “back-to-Africa” gene flow more than 12,000 years ago (ya), prior to the Holocene. The indigenous North African ancestry is more frequent in populations with historical Berber ethnicity. In most North African populations we also see substantial shared ancestry with the Near East, and to a lesser extent sub-Saharan Africa and Europe. To estimate the time of migration from sub-Saharan populations into North Africa, we implement a maximum likelihood dating method based on the distribution of migrant tracts. In order to first identify migrant tracts, we assign local ancestry to haplotypes using a novel, principal component-based analysis of three ancestral populations. We estimate that a migration of western African origin into Morocco began about 40 generations ago (approximately 1,200 ya); a migration of individuals with Nilotic ancestry into Egypt occurred about 25 generations ago (approximately 750 ya). Our genomic data reveal an extraordinarily complex history of migrations, involving at least five ancestral populations, into North Africa.

## Author Summary

Proposed migrations between North Africa and neighboring regions have included Paleolithic gene flow from the Near East, an Arabic migration across the whole of North Africa 1,400 years ago (ya), and trans-Saharan transport of slaves from sub-Saharan Africa. Historical records, archaeology, and mitochondrial and Y-chromosome DNA have been marshaled in support of one theory or another, but there is little consensus regarding the overall genetic background of North African populations or their origin and expansion. We characterize the patterns of genetic variation in North Africa using ∼730,000 single nucleotide polymorphisms from across the genome for seven populations. We observe two distinct, opposite gradients of ancestry: an east-to-west increase in likely autochthonous North African ancestry and an east-to-west decrease in likely Near Eastern Arabic ancestry. The indigenous North African ancestry may have been more common in Berber populations and appears most closely related to populations outside of Africa, but divergence between Maghrebi peoples and Near Eastern/Europeans likely precedes the Holocene (>12,000 ya). We also find significant signatures of sub-Saharan African ancestry that vary substantially among populations. These sub-Saharan ancestries appear to be a recent introduction into North African populations, dating to about 1,200 years ago in southern Morocco and about 750 years ago into Egypt, possibly reflecting the patterns of the trans-Saharan slave trade that occurred during this period.

## Introduction

The census size of Mediterranean North Africa exceeds 160 million people [1], but relatively little is known about the genetic makeup of these populations and the demographic history of migration between North Africa and neighboring regions. Mediterranean North Africans are often grouped with Near Eastern populations because populations in both regions speak primarily Afro-Asiatic languages, like Arabic, and phenotypic attributes, like lighter skin pigmentation, differentiate many North Africans from sub-Saharan Africans. Recently, geneticists have attempted to replicate disease associations identified in Europeans and Near Eastern groups with North African populations, reflecting a hypothesis of shared genetic ancestry, with mixed results [2]–[5]. In this paper, we present analysis of autosomal single nucleotide polymorphism (SNP) array data for seven North African populations (see Materials and Methods), distributed along an east-to-west transect across the continent. We clarify the population structure of North Africa and explicitly interrogate the history of gene flow into North Africa from the Near East, Europe and sub-Saharan Africa.

Prior genetic studies, largely from uniparentally inherited markers, have not resolved the location origin of North African populations or the timing of human dispersal(s) into North Africa. Analyses based on the frequencies of a small number of autosomal genetic polymorphisms and uniparental markers have shown that the genetic landscape follow an east-west pattern with little to no difference between Berber- and Arab-speaking populations [6], [7]. Mitochondrial data, for example, indicate an early back-to-Africa migration [8]–[10], but Y-chromosome markers largely support a Neolithic expansion and historic period gene flow throughout the Mediterranean [11] (though see [12]). Do current North Africans retain genetic continuity with the first modern human occupants of northern Africa from more than 50,000 years ago (ya) or was northern Africa primarily repopulated during the Holocene by herding and farming populations from elsewhere? Evidence of Neolithic migration from the Near East is supported by the introduction of domestic animals like cows, sheep and goats to North Africa. But the indigenous development of ceramics in Saharan Africa by 9,000 ya is also suggestive of an incipient form of agriculture or pastoralism, prior to any demic diffusion from the Near East [13].

Less controversial is the observation that many North African populations now speak Arabic and that this language shift occurred primarily after the Arabic conquest 1,400 ya. This Arabic shift is well documented, but it remains unknown how deeply recent migrations (<2,000 ya) from the Arabian and Iberian Peninsulas shaped the genetic diversity of current North African populations. In addition, sub-Saharan influence has been detected in North African samples by all types of genetic markers analyzed, although it is unknown how recent this gene flow might have been [14]–[16]. Initial autosomal SNP analysis of the Algerian Mozabites indicated they carry ancestry from Europe, the Near East and sub-Saharan Africa; neighbor-joining phylogenetic analysis suggested that Mozabites branch off with Out-of-African populations, but are an outgroup to all Near Eastern populations in the Human Genome Diversity Panel (HGDP-CEPH) [17]. In short, the origins of North African populations and the number of subsequent migrations from neighboring regions have been poorly resolved.

Genomic models of admixture in human populations have largely been confined to cases of two-way admixture such as African-American [18]–[20] and some Hispanic-Latino groups such as Mexican-Americans [21]–[23]. However, a two-population model is likely inappropriate for North African populations (as it is for some Caribbean groups such as Puerto Ricans [24]) given multiple putative migrations proposed in earlier studies. Moreover, while African-Americans and Hispanic/Latino have ancestries from highly divergent source populations, North Africans may have ancestry from more closely related populations, for example Europeans and Near Easterners. We extend a principal component analysis-based (PCA) method of local ancestry assignment [20] in order to allow for three possible ancestral populations. With haplotypes for various ancestries inferred from PCA-based assignment, we model the time and mode of migrations from neighboring regions into North Africa.

## Results

### Population Structure across North Africa

**Figure 1:**
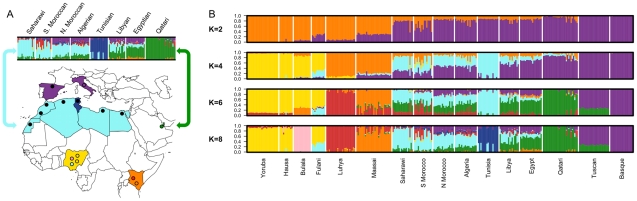
Map of samples and population structure of North Africa and neighboring populations. A) On the map, star symbols indicate the location of new population samples and circles indicate previously published samples. The decreasing proportions of Maghrebi ancestry is indicated in a west-to-east gradient of blue across North Africa. An unsupervised clustering algorithm, *ADMIXTURE* [25] was used to analyze population structure among 13 African, 2 European and 1 Near Eastern populations based on approximately 300 K autosomal SNP loci in common. The two main gradients of ancestry in North Africa (at *k* = 8), Maghrebi and Near Eastern, are emphasized with arrows. Other population colors in open circles match the colors displayed in the population structure analysis. B) We plot the full dataset assuming *k* = 2,4,6,8 ancestral populations. *k* = 10 and log likelihoods are presented in Figure S1.

**Figure 2:**
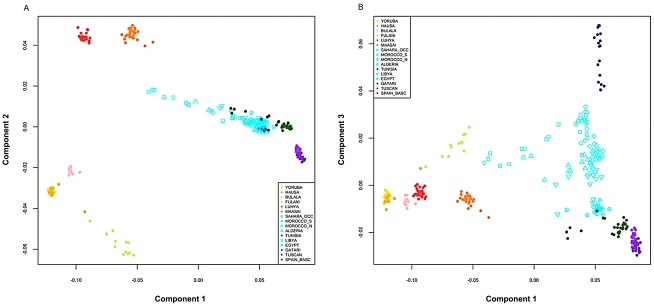
Multidimensional scaling components discriminating genetic clusters in Africa. We used multidimensional scaling (MDS) to discriminate clusters of genetic variation within Africa and neighboring regions. MDS was applied to the pairwise, individual identity-by-state (IBS) matrix of 279,500 SNPs using PLINK 1.07 software [45]. The top three MDS components were plotted together using R 2.11.1. A) In axis one is plotted dimension 1 and in axis two is plotted dimension 2. B) In axis one is plotted dimension 1 and in axis two is plotted dimension 3. Population colors match Figure 1B (*k* = 8), with the exception that the Fulani group was highlighted as a distinct population, indicated in light green. North African populations are all indicated in turquoise except for Tunisians that are shown in dark blue.

In order to characterize population structure across North Africa, we combined our genotype data for the seven North African populations with population samples from western Africa, eastern Africa, Europe and the Near East (see Materials and Methods). A representative subset of these samples is displayed in Figure 1 and Figure 2. We applied both classic multidimensional scaling (MDS) with an LD-reduced set of 280 K SNPs on the identity by state (IBS) matrix and an unsupervised clustering algorithm, *ADMIXTURE* [25], to explore patterns of population structure. In *ADMIXTURE*, we explored *k* = 2 through 10 ancestral populations to investigate how assumptions regarding *k* impact our inference of population structure in North Africa. Log likelihoods for successively increasing levels of *k* continue to increase substantially as *k* increases (Figure S1B). However, visualization of *k* = 10 indicates that very high order clusters pulled out related individuals in the Tunisian Berber sample (Figure S1); for this reason we focus on *k* = 2 through 8.

Our North African population samples are clearly differentiated from other African populations (Figure 1, Figure 2). MDS component 1 separates sub-Saharan Africans from populations that currently reside outside of Africa (OOA), and the North African populations cluster closest to the Near Eastern Qatari. A subset of individuals are intermediate between the North and sub-Saharan African samples (Figure 2, Figure S2). At *k* = 2 (*ADMIXTURE*), 80% of the ancestry in North African individuals is assigned to a cluster defined by its maximum frequency in Near Eastern and European populations (Figure 1). MDS component 2 differentiates western from eastern sub-Saharan Africans (Figure 2A). MDS component 3 differentiates populations thought to have a high degree of autochthonous ancestry (i.e. Tunisian Berbers and Saharawi) from populations outside of Africa. Interestingly, the MDS component 3 appears to be largely independent of the amount of sub-Saharan ancestry (Figure 2B) and North Africans are dispersed along the MDS component 3 axis, with the Tunisian Berbers occupying the extreme end of this gradient.

A gradient also appears in the higher *k* ancestral population plots of the *ADMIXTURE* analysis (Figure 1). Assuming 4 or more ancestral populations (*k* = 4 through 10, Figure S1) there is a cline of putative autochthonous North African ancestry decreasing in frequency from Western Sahara eastward to Egypt. We refer to this North African ancestral component as the “Maghrebi” throughout the remainder of the paper, reflecting the primary geographic distribution of this ancestry in the Maghreb: West Sahara, Morocco, Algeria and Tunisia. The west-to-east decline in Maghrebi assignment is only interrupted by the Tunisian Berbers, who are assigned nearly 100% Maghrebi ancestry. The Tunisian Berbers further separate as a distinct population cluster at *k* = 8. An opposite cline of ancestry appears to originate in the Near East (i.e. Qatari Arabs) and decreases into Egypt and westward across North Africa (*k* = 6, 8).

At *k* = 6 through 8, all North African populations except for Tunisians have sub-Saharan ancestry, present in most individuals, though this ancestry varies between 1%–55%. Interestingly, eastern populations (i.e. Libya and Egypt) share ancestry assigned to both the Bantu-speaking Luhya and the Nilotic-speaking Maasai, whereas western populations share ancestry mainly with the Luhya. Of note is that the South Moroccan and western Saharan populations contain considerable variation across individuals in the amount of sub-Saharan ancestry (see also [14], [26]), consistent with recent admixture.

### Divergence between North Africans and Neighboring Populations

**Figure 3:**
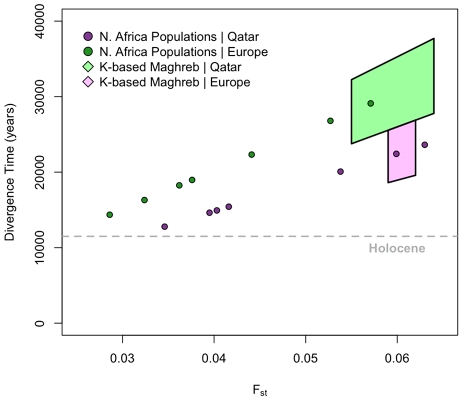
Divergence time estimates among European, Maghrebi, and Near Eastern ancestral populations. We use population-based F_st_ to estimate divergence time between each of the North African populations and the Qatari (green dots) and Tuscans (purple dots), respectively. We assume point estimates of effective population sizes based on autosomal haplotype heterozygosity estimates from Li et al. [17] (*Material and Methods*). We further estimate ancestral population clusters assigned at *k* = 5–8 with ADMIXTURE. Assuming that the ancestral clustering procedure has removed, or at least mitigated, the effect of recent migrations into Mediterranean populations, we then use F_st_ to estimate divergence times between these ancestral clusters. The range of *k*-based estimates for Maghrebi versus Near Eastern ancestry is indicated with light green polygon. The range of *k*-based estimates for Magrebi versus European ancestry is indicated with light purple polygon.

We estimate F_st_ by comparing each of the North African populations to the Tuscans and Qatari respectively. Estimates range between 0.035–0.063 (Figure 3). In order to quantify population divergence among these groups, we use the relationship between F_st_ and the effective size “*N_e_*” to estimate the divergence time “*t*” (see Materials and Methods). Since this model neglects migration, we expect our results to form a *lower* bound on the population divergence time, as similar levels of population divergence would require a longer separation in the presence of migration. Additionally, the model assumes that populations have had similar demographic histories (i.e. if there was a bottleneck, all populations were affected equally); as all populations derive the majority of their ancestry from an Out-of-Africa ancestral population, and the OOA bottleneck is the primary signature in OOA populations, we believe this assumption is valid. Estimates for population *N_e_* were taken from Li et al. [17]. All estimates of population divergence between the North Africans and the European/Near Eastern samples predate the Holocene.

We then attempted to obtain more accurate estimates of divergence time by controlling for recent migration. We calculated a second set of F_st_ estimates using *cluster-based* allele frequencies from ADMIXTURE among the Maghrebi, European and Near Eastern ancestries, when we considered higher order *k* = 5∶8 ancestral clusters. As indicated in Figure 3, population divergence between the Maghrebi and the European and Near Eastern populations occurred between 18,000–38,000 ya. The bounds here represent variation in ancestral *k* estimates and assumptions regarding *N_e_*, as Near Eastern populations have a greater estimated *N_e_* than European. Although these divergence time estimates may not be precise, as they do not adequately model ancient migration, they do suggest that the population divergence between the ancestral Maghrebi population and neighboring Mediterranean populations occurred at least 12,000 ya and indeed more likely predated even the Last Glacial Maximum.

### Within Population History

**Figure 4:**
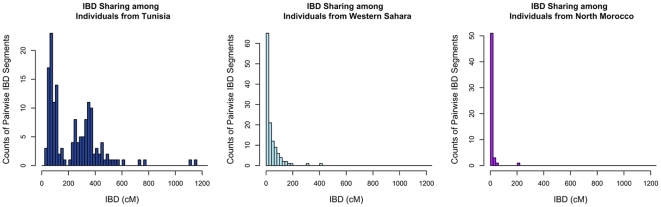
Distribution of long segments that are identical by descent (IBD) for pairs of individuals. The cumulative amount of DNA shared IBD between each pair of individuals within a population was summed. We display counts of the number of pairs sharing cumulative IBD in A) Tunisian Berbers, B) Saharawi and B) North Moroccans.

Given the complex patterns of admixture apparent from population structure analyses, we asked if populations differed in the proportion of DNA that individuals within populations shared identically. We estimate the amount of DNA shared identically by descent (IBD) using the GERMLINE software [27], with a 5 cM threshold to eliminate false positive IBD matches. The estimated cumulative amount of IBD between pairs of individuals within each population is illustrated in Figure 4 with the Tunisians, Saharawi, and North Moroccans. Most of our North African populations shared little IBD or displayed an exponential-like decline in the cumulative amount of IBD, indicating that the great majority of individuals in these populations were only distantly related (i.e. had less IBD than predicted in third cousins). However, the Tunisian Berber population displayed an excess of pairs of individuals sharing 200–1200 cM IBD. This bimodal distribution indicates that many 1^st^ and 2^nd^ cousin genetic equivalent pairs were present in this sample, even though donors declared themselves to be unrelated during the sampling process. Analysis of long runs of homozygosity (ROH) indicate that the Tunisian population averaged almost twice as much of their genome is in ROH than other North African populations, 230 Kb versus 120 Kb respectively (Figure S3). The pattern of ROH and pairwise IBD in the Tunisian Berbers is likely the result of endogamy due to geographic isolation or cultural marriage preferences.

### Local Ancestry Assignment across the Genome

Our cluster-based analysis identified five distinct ancestries in North Africa that we refer to as: Maghrebi, European, Near Eastern, eastern and western sub-Saharan Africa. In order to test whether sub-Saharan African ancestry was an ancient or recent migration signature, we considered the length of sub-Saharan haplotypes. First, to assign local, ancestry-specific haplotypes across a genome, we implemented a new principal component-based admixture deconvolution approach (PCADMIX) for three ancestral populations (see Materials and Methods, Figure S4) [20], [28]. We focus on admixed populations at either end of North Africa, specifically our population samples of South Moroccans and Egyptians.

**Figure 5:**
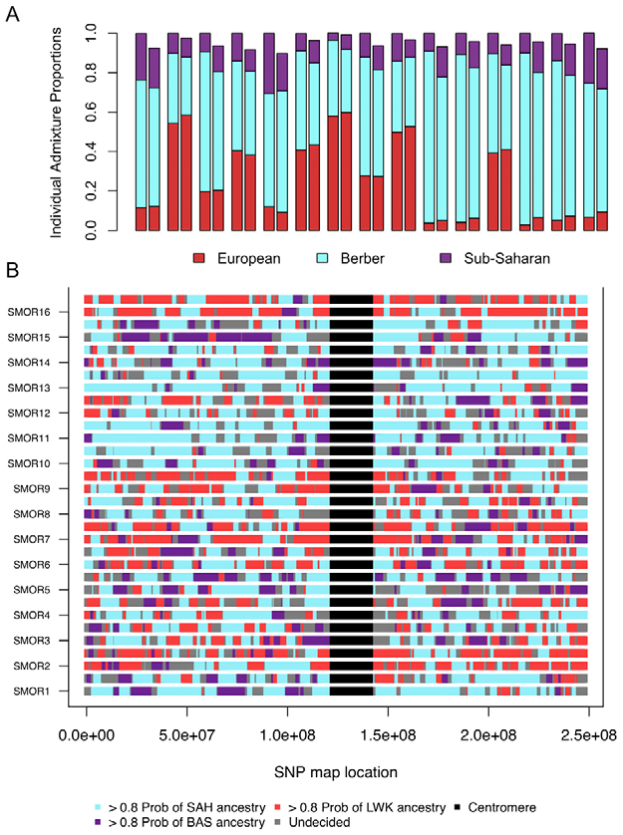
Correlation between ancestry proportions inferred from ADMIXTURE and PCADMIX. We compare the proportions of ancestry inferred from assuming 3 ancestral populations in individuals from South Morocco A) using a clustering algorithm set to *k* = 8 (Figure 1) summing the sub-Saharan ancestry, both Qatar and European ancestry, and Maghrebi ancestry. We compared these estimates (left bar) to our PCA-based local ancestry assignment estimates (right bar). The three ancestral populations were Saharawi [SAH], Bantu-speaking Luhya [LWK], and Spanish Basque [BAS]. B) Genome admixture deconvolution on chromosome 1 of sixteen South Moroccans. Using a principal component-based method of admixture deconvolution, we assign local ancestry to South Moroccan individuals. We implement our PCA-based method for k = 3, and choose the ancestral populations based on the three ancestral populations were Saharawi [SAH], Bantu-speaking Luhya [LWK], and Spanish Basque [BAS]. Chromosome 1 for all sixteen South Moroccans is presented for both the maternal and paternal haplotypes.

PCADMIX requires predefined ancestral groups. For this purpose, we assume South Moroccans have ancestry from three primary sources: Maghrebi ancestry (e.g. Saharawi), eastern Bantu-speakers (e.g. Luhya) and European (e.g. Spanish Basque) (Figure 5A). We similarly assume Egyptians have ancestry from four primary source populations: Maghrebi (e.g. Saharawi), eastern Nilotic-speakers (e.g. Maasai), Near Eastern Arabs (e.g. Qatari) and European (e.g. Spanish Basque). These source populations reflect the ancestry assigned in our clustering algorithm analysis (Figure 1). According to our *ADMIXTURE* results, two distinct sub-Saharan ancestries are present in Egyptian individuals at *k* = 6∶10; these two ancestry components are highest in the Kenyan Luhya and Maasai populations. However, the “Luhya” ancestry is present at very low proportions, below 10% at *k* = 6 and below 5% at *k* = 8 and there is also “Luhya” ancestry detectable in Maasai populations. Thus, we chose the Maasai as the best ancestral sub-Saharan population for extant Egyptians.

If our choice of source populations for an admixed individual is accurate (i.e. the source populations are reasonable representatives of an ancestral population) we expect similar estimates of ancestry proportions between PCADMIX-ADMIXTURE when ancestry in PCADMIX is assigned with a posterior probability threshold of 0.8 (, Figure 5, Figure S5). We used the Saharawi as our proxy Maghrebi population, since the high relatedness in the Tunisian samples is likely to cause reduced ability to infer Maghrebi tracts in more diverse populations. Our sample of Tunisian Berbers retains the highest amount of Maghrebi ancestry, without substantial evidence of admixture with sub-Saharan, European or Near Eastern populations. However, their bimodal mean IBD distribution (Figure 4A) indicates a high proportion of 1^st^–2^nd^ cousin equivalents and suggest that our sample of Tunisian Berbers comes from an isolated, endogamous population with diversity that is likely reduced relative to other Maghrebi populations. Thus, although their low degree of non-Maghrebi admixture might make them ideal as a Maghrebi source population, reduced haplotypic diversity means that we are likely to under-call true Maghrebi segments from other, more diverse populations. This expectation was borne out in our PCA-based admixture deconvolution of southern Moroccans when comparing Tunisian versus Saharawi as a Maghrebi source population (Figure S6). We note that when using either the Tunisian Berbers or the Saharawi, the Maghrebi component in other individuals (e.g. Egyptian, South Moroccan) tended to be underestimated in comparison to the ADMIXTURE proportions (Figure 5A, Table S2). We also infer independent admixture proportions in the Algerian, South Moroccan and Saharawi samples by running LAMP [29] to estimate local ancestry using 3 source populations: Tunisians, Basque and Luhya; with LAMP we also observe a likely excess of inferred European ancestry in the Algerian, South Moroccan and Saharawi samples (7).

### Migration Parameters

The length of tracts assigned to distinct ancestries in an individual is informative regarding the time and mode of migration from one ancestral population into another. After a migrant chromosome enters a population, the length of the migrant ancestry tract is broken down over time due to the process of recombination. We use a maximum likelihood approach developed by Pool and Nielsen [30] to estimate the time of change in migration rate between populations based on the length and number of migrant tracts in the absorbing sink (or “admixed”) population.

**Figure 6:**
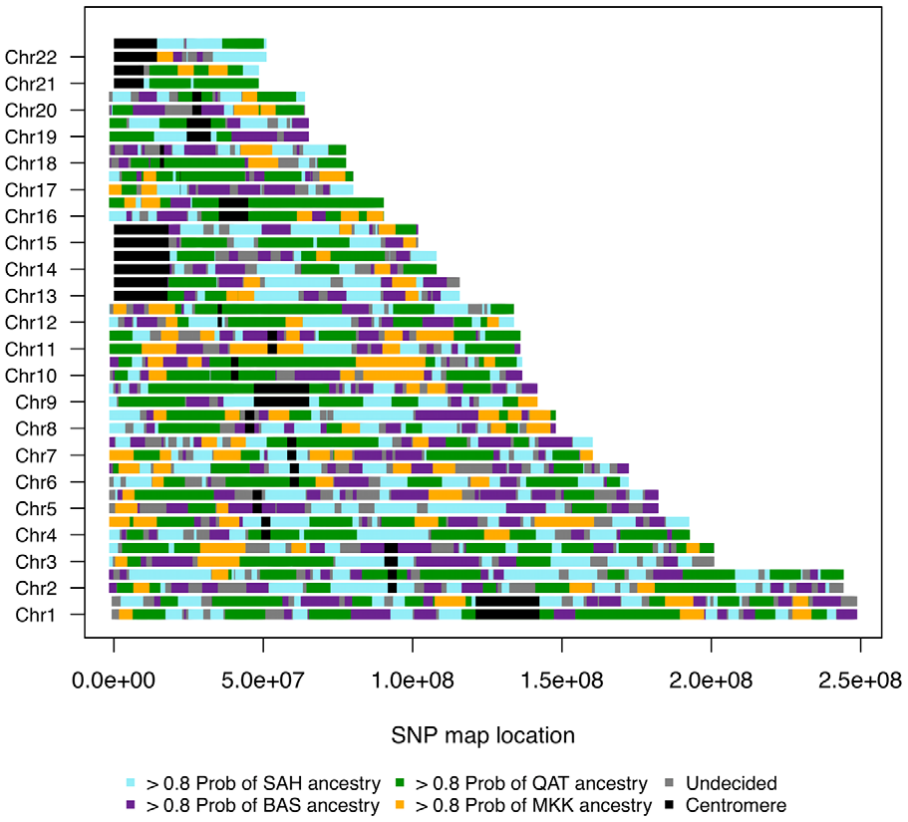
Genome admixture deconvolution karyogram of an Egyptian. A single Egyptian individual is presented for ancestry assuming *k* = 4 source populations: Saharawi [SAH], Nilotic-speaking Maasai [MKK], Spanish Basque [BAS] and Arabic Qatari [QAT]. Maasai segments (which were inferred from *k* = 3 and were highly diverged from the SAH, QAT, BAS segments) are layered on top of the inferred Maghrebi/Qatari/Basque ancestral karyogram, for *k* = 4 putative source populations.

We first consider a continuous migration model where migration occurs at a constant rate from *T* generations ago to present day. We assume that there has been no migration between the source and sink populations prior to the initial time 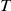 of a migration into the admixed population. We tabulate the number of migrant tracts in the Moroccans and Egyptians, where each migrant tract has a posterior probability >0.8. To reduce biases due to our lower sensitivity to short tracts, we only modeled tracts longer than 3 cM, and considered assigned tracts with posterior probability >0.8. With a 3 cM cutoff we expect to capture 50% of tracts from 55 generations ago and 10% of tracts from 130 generations ago (see Materials and Methods). Unassigned short tracts (i.e. the “undecided” regions, Figure 5B and Figure 6) within a long continuous migrant segment can be artificially shortened by spikes of low posterior probability. Unassigned tracts that were situated within a tract of one ancestry and which maintained a posterior probability >0.5 for the same neighboring ancestry were considered to as one long ancestry tract.

We focus on the sub-Saharan African migrant tracts in South Moroccans (Figure 5B) and Egyptians (Figure 6). These tracts tend to be highly diverged from other ancestries in the population (F_st_>0.10) and populations with similar divergence resulted in accurate haplotype assignment in prior testing [28]. Under a model of constant migration from the Bantu-speaking Kenyans and southern Moroccans started about 41 generations ago (*ga*) (95% CI: 39–44*ga*) assuming there was no migration occurring prior to this period. The confidence interval calculations, obtained by resampling sub-Saharan migrant tracts with replacement, do not take into account possible biases caused, for example, by the model assumption of a fixed migration rate.

### Constant Versus Episodic Migration

We hypothesized that the distribution of sub-Saharan African tracts in the Moroccans and Egyptians might better reflect a single episode or “pulse” of migration. In order to test this hypothesis, we modify Pool and Nielsen's [30] approach to conform to a pulse model (see Materials and Methods). We compared the log likelihoods summed over all migrant tracts under constant and pulse migration models for each population maximized over the relevant parameters, and present the model with the higher log likelihood (Table 1). Estimates of the time of migration are more recent under a pulse model. The younger estimate occurs because the model fit must account for relatively long migrant tracts in the data; under a constant migration model these tracts represent recent migrants, but for a single episode of migration, long tracts can only be accounted for by recent migration of the entire sample. In order for the average migrant tract length to be equal in the two models, migration must have started more than twice as long ago in the constant migration model compared to the pulse model (Table 1). Our Egyptian sample of Nilotic segments (derived from Maasai) has a better log likelihood under a pulse migration model, estimated as time since admixture of 24*ga* (95% CI: 23–26*ga*) rather than 51*ga* under a constant migration model (Table 1).

**Table 1:** Models of migration into North Africa.

Admixed Population	Migrant Population^1^	Migration Model^2^	Log Likelihood	Time of migration (*G*)^3^	Bootstrap 95% CI^4^
Egyptians	Maasai (Kenya)	Continuous	2682	51	47.7–55.8
		**Pulse**	**2705**	**24**	**22.8–25.8**
South Moroccans	Luhya (Kenya)	**Continuous**	**5386**	**41**	**39.1–44.0**
		Pulse	5365	19	18.1–19.7

1 Segments from the migrant population were required to be greater than 3 cM in length. Given this minimum threshold, 815 migrant Egyptian and 1,275 migrant South Moroccan segments were discarded.

2 Two migration models were tested: a “pulse” model assumes a single episode of migration occurred at T_0_ followed by no further migration, a “continuous” model assumes constant migration from T_0_ to the present day. Log likelihoods given either model were compared and we present the model with the highest log likelihood.

3 The maximum likelihood estimate of time since migration initially began “T_0_” from the migrant population into the admixed: population. We assume prior migration between the populations was zero. Time since migration began T_0_ is indicated in generations.

4 The 95% confidence interval was estimated by sampling migrant Egyptian segments (n = 1,246) and migrant South Moroccan segments (n = 2,770) 1,000 times with replacement.

## Discussion

### Out of Africa and Back Again?

By sampling multiple populations along an approximate transect across North Africa, we were able to identify gradients in ancestry along an east-west axis (Figure 1 and Figure 2). Notably, even northwestern populations with very high proportions of Maghrebi ancestry, such as the Tunisians and Saharawi, still cluster with Out-of-Africa populations in the population structure analyses (Figure 1 (*k* = 2), Figure 2). This observation of clustering formed the basis for further analyses to distinguish between two alternative demographic models. First, North Africans could be closer to OOA populations due to extensive gene flow, likely from the Near East, over the past ∼50 Kya. Second, North Africans could be closer to OOA populations if the two groups had diverged more recently than either had split with sub-Saharan Africans.

We can reject a simple model of long-term continuous gene flow between the Near East and North Africa, as evidenced by clear geographic structure and non-zero F_st_ estimates. F_st_ estimates between the inferred Maghrebi cluster and sub-Saharan Africans are two to three-times greater than F_st_ between the Maghrebi and Europeans/Near Easterners ancestral clusters (Table S3). We then address whether this population structure was recent or ancient. Although F_st_ estimates from ascertained data may be biased, as rare alleles are under-represented in the site frequency spectrum, comparison of African-European F_st_ from resequencing data and the Affymetrix 500 K platform showed only a negligible difference [31]. Assuming reasonable effective population sizes for North African Maghrebi and neighboring populations [17], we first showed that all North African populations are estimated to have diverged from OOA groups more than 12,000 ya (Figure 3). After accounting for putative recent admixture (Figure 1), the indigenous Maghrebi component (*k*-based) is estimated to have diverged from Near Eastern/Europeans between 18–38 Kya (Figure 3), under a range of N_e_ and *k* values. We hence suggest that the ancestral Maghrebi population separated from Near Eastern/Europeans prior to the Holocene, and that the Maghrebi populations do *not* represent a large-scale demic diffusion of agropastoralists from the Near East.

With model parameters for divergence approximately estimated, we then ask whether North African ancestral populations were part of the initial OOA exit and then returned to Africa [8], or if an *in situ* model of population persistence for the past 50 Kya is more likely (with variable episodes of migration from the Near East)? We can address this question only indirectly with contemporary samples; however, several auxiliary observations point toward the former hypothesis. Substantially elevated linkage disequilibrium in all of these North African population samples, compared to sub-Saharan populations [32], is consistent with a population bottleneck. Hellenthal et al. [30] also observed that the reduction in the number of haplotype founders required to reconstruct the Mozabite population, as compared to other African populations, could be explained by a population bottleneck. If North African ancestral populations persisted *in situ*, then we need to invoke two population bottlenecks, one in the ancestors of North Africans (including the Berbers) and one for OOA groups. Alternatively, the “OOA” bottleneck would need to occur in North Africa, rather than when groups moved out of the continent [33]. The second possibility appears at odds with most published models of the movement of modern humans outside of Africa.

A scenario where North African Maghrebi ancestry is the result of *in situ* population absorbing Near Eastern migrants would likely need the following premises to explain the results here and elsewhere: a) an Out-of-Africa migration [concurrent with bottleneck] occurs 50–60 Kya, geographically dividing North African and Near Eastern populations; b) North Africans experience a separate bottleneck; c) gene flow maintains similarity between the two geographically distinct populations; d) the gene flow then ceases or slows roughly between 12–40 Kya in order to allow sufficiently distinct allele frequency distributions to form. In contrast, we find it more parsimonious to describe model where: a) an OOA migration occurs [concurrent with a bottleneck]; b) OOA populations and North Africans diverge between 12–40 Kya when a migration back-to-Africa occurs. These models should be further tested with genomic sequence data, which have better power to detect magnitude and timing of bottlenecks, and to estimate the true joint allele frequency spectrum.

More recently, the substantial, east-to-west decline of Near Eastern ancestry (Figure 1A) could represent a defined migration associated with Arab conquest 1,400 ya or merely gene flow occurring gradually among neighboring populations along a North African | Arabian Peninsula transect. Although we observe a declining amount of Maghrebi ancestry from northwest-to-northeast, it is possible that other geographically North African samples (e.g. Egyptians further south than the sampled Siwa Oasis) do not conform to this geographic cline. Finally, we also observe European ancestry that is not clearly accounted for by the inclusion of a Near Eastern sample. Additional migration coming from Europe might be plausible, though the origin and the period where it took place cannot be determined with the present data. The less than 25% European ancestry in populations like Algerians and northern Moroccans could trace back to maritime migrations throughout the Mediterranean [34]. Alternatively, the Qatari could represent a poor proxy for an Arabic source population, causing additional diversity to be assigned European (e.g. European ancestry tracts were not reliably assigned as European with PCADMIX).

In summary, although paleoanthropological evidence has established the ancient presence of anatomically modern humans in northern Africa prior to 60,000 ya [35], the simplest interpretation of our results is that the majority of ancestry in modern North Africans derives from populations outside of Africa, through at least two episodes of increased gene flow during the past 40,000 years (Figure 1, Figure 2, Figure 3).

### Reconstructing Multiple Admixed Ancestries

Multiple local ancestry assignment methods, including PCADMIX, require thinning genotype datasets to remove alleles in high linkage disequilibrium between populations [29], [36]; this step discards information regarding haplotype patterns that tend to be more informative than genotypes when using data biased by SNP ascertainment [37]. HAPMIX incorporates both LD information and uncertainty in phase inference for haplotypes [18], but the software is currently limited to a two-population model. Our ancestral proportions of European and sub-Saharan ancestry for many North Africans at *k* = 2 (Figure 1) are similar to those obtained with HAPMIX by Price et al. [18] for the HGDP Algerian Mozabites, assuming a two-population mixture of northern Europeans and Yoruba. However, our results show that increasing the number of possible ancestral populations reveals multiple, diverse ancestries (e.g. Maghrebi, Near Eastern, Nilotic) and that the proportion of sub-Saharan African assignment decreases as these other ancestries are accounted for. This decrease in assigned sub-Saharan ancestry in North African samples, from a *k* = 2 model, is consistent with an interpretation that Maghrebi or Near Eastern diversity that is not present in the panel populations is more likely to be assigned to the more diverse, Sub-Saharan African ancestry. Using a two-population admixture model, Price et al. [18] estimated the time of migration from sub-Saharan Africa into the Mozabites to have begun about 100 generations ago (or more). Our results suggest that sub-Saharan African and Maghreb admixture is considerably more recent, 24–41 generations ago (and even the upper 95% CI estimate under either model is 55*ga*, Table 1). The discrepancy between these two estimates may result from our incorporation of multiple source populations, our use of non-linear models to estimate migration timing and the elimination, in Price et al. [18], of individuals with megabase long African segments.

### Time of Migration Estimation

We use a two-population model of migration where we measure the number and length of migrant tracts observed in the admixed population. However, as argued earlier, North African populations have absorbed migrants from multiple episodes of migration. We use three- and four-population admixture deconvolution to identify the tracts from these separate migrations. One complication with this approach is the possibility that source populations that contribute migrants to North Africa are *themselves* exchanging migrants. For example, Near Eastern populations expanded into European continent during the Neolithic, and even an isolated population like the Spanish Basque may have genomic segments that trace back to the Neolithic expansion [38], [39]. In this case, estimation of the time of migration of Arabic individuals into North Africa would be biased by Basque segments of Arab ancestry that were contributed by Europeans, but are locally assigned to Arabic ancestry. We confine our migration estimates to those from sub-Saharan populations into North Africans because there has likely been relatively little recent gene flow between sub-Saharan Africans and the European/Near Eastern populations. Moorjani et al. [40] present evidence for recent gene flow (<100 generations ago) from Africa to the Near East and Europe. But, they hypothesize it might be due to North African migrations, rather than sub-Saharan Africa.

### Migration Implications

Assuming a 30-year generation time [41], the proposed migration of sub-Saharans to southern Morocco at about 1,200 years ago coincides with the rise of the Ghana Empire, involved in the trans-Saharan slave trading, and the “Great Berber Uprising” which established Berber kingdoms throughout Morocco. We use a Bantu-speaking population from Kenya as a source population for this migration, as North African individuals with sub-Saharan ancestry appeared to be closer to the Luhya than the Nigerian Yoruba (Figure 1, Figure 2 and Figure S2). However, there are likely other western African populations genetically similar to Kenyan Bantu-speakers. We do not interpret this association as an explicit migration from Kenya to southern Morocco. We also use the length of Nilotic tracts in Egyptians to ask if sub-Saharan ancestry (apparent in Figure 1 and Figure 6) also appears to be a recent introduction. Under a pulse model of migration, a significant increase in gene flow likely occurred ∼700 ya, after the Arabic expansion into North Africa 1,400 ya. Our migration results are in agreement with previous studies based on mtDNA analysis where gene flow into eastern and western North Africa appeared to have different sub-Saharan population sources [10], [16].

### Conclusion

Our genome-wide dense genotyping data from seven North African populations allow us to address outstanding questions regarding the origin and migration history of North Africa. We propose that present-day ancestry in North Africa is the result of at least three distinct episodes: ancient “back-to-Africa” gene flow prior to the Holocene, more recent gene flow from the Near East resulting in a longitudinal gradient, and limited but very recent migrations from sub-Saharan Africa. Population structure in North Africa is particularly complex, and future disease or phenotypic studies should carefully account for local demographic history. However, the rich history of gene flow can also help empower genome-wide association mapping via admixture mapping techniques [42]. For example, the variable but relatively long haplotypes of sub-Saharan ancestry are amenable to admixture mapping approaches developed for African-American samples. In conclusion, North African populations retain a unique signature of early “Maghrebi” ancestry, but North African populations are not a homogenous group and most display varying combinations of five distinct ancestries.

## Materials and Methods

### Samples and Data Generation

A total of 152 individuals representing seven different North African locations and the Basque Country were included in the present study. Informed consent was obtained from all of them. Samples were genotyped on the Affymetrix 6.0 chip, and after quality control filtering for missing loci and close relatives, 125 individuals remained: 18 from North Morocco, 16 from South Morocco, 18 from Western Sahara, 19 from Algeria, 18 from Tunisia, 17 from Libya and 19 from Egypt. Further information on the samples may be found in Table S1. Moreover, 20 individuals from the Spanish Basque country were included in the analysis. Data are publicly available at: bhusers.upf.edu/dcomas/. In order to study the population structure and the genetic influence of migrants in the region a database was built including African and European populations from HapMap3 [43], western Africa [20], and 20 Qatari from the Arabian Peninsula [44] as Near Eastern representatives. Written informed consent was obtained from the participants and analyses were performed anonymously. The project obtained the ethics approval from the Institutional Review Board of the institution involved in the sampling (Comitè Ètic d'Investigació Clínica - Institut Municipal d'Assistència Sanitària (CEIC-IMAS) in Barcelona, Spain).

### Population Structure

An unsupervised clustering algorithm, *ADMIXTURE* [25], was run on our seven new North African populations, Spanish Basque, Near Eastern Qatari, western Africans, HapMap3 Kenyan Luhya, Maasai and Italian Tuscans. Nine ancestral clusters (*k* = 2 through 10) in total were tested successively. Log likelihoods for each *k* clusters are available in Figure S1B. F_st_ based on allele frequencies was calculated in ADMIXTURE for each identified cluster at *k* = 8. Given the high heterogeneity in Qatari population, we present individuals with the lowest sub-Saharan, European and North African ancestries and higher Near Eastern ancestry, based on ADMIXTURE. Multidimensional scaling (MDS) was applied to the pairwise IBS Matrix of 279,528 SNPs using PLINK 1.07 software [45]. The top three MDS components were plotted together using R 2.11.1. Population divergence estimates from the cluster-based allele frequencies from ADMIXTURE (*k* = 5–8) were obtained using [46]:
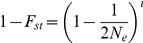
The cluster-based allele frequencies will be less biased by recent migration between populations. Estimates of population divergence, though potentially older if migration is unaccounted for in the F_st_ estimate, are unlikely to be younger if the range of *N_e_* sizes is realistic.

### Phasing

In previous work, imputation accuracy was tested in a sample of Algerian Mozabites and other populations from the Human Genome Diversity Project (HGDP-CEPH) [37]. Among all the African populations, the Mozabites had the poorest imputation accuracy when the sub-Saharan Yoruban sample was used to predict allele states [37]. For this reason, we used multiple populations for phase inference. North African, Qatari and Basque genotypes were phased using BEAGLE 3.0 software [47]. Phased haplotypes from three HapMap3 populations (i.e. Maasai, Yoruba, and Tuscans) were used as seeds for haploype inference; each HapMap3 population was randomly sub-sampled for 30 individuals each in order to prevent over-representation of haplotypes from a single geographic region. The Basque, Qatari and all North African populations were phased with the same three seed populations to prevent discrepancies based solely on different haplotype seeds.

### Inference of IBD

We estimate the amount of DNA shared identically by descent (IBD) using the GERMLINE software [27], with a 5 cM threshold to eliminate false positive IBD matches. All 5 cM or greater segments shared IBD between pairs of individuals were summed, and histograms created for sharing within each North African population.

### PCA–Based Local Ancestry Assignment

Local ancestry was assigned with a new PCA-based method, PCADMIX. This method uses *phased* genotype data (i.e., haplotypes) to determine exact posterior probabilities along each chromosome. PCADMIX relies on Principal Components Analysis (PCA) to quantify the information that each SNP contributes to distinguishing the ancestry of a genomic region. PCADMIX is publicly available at sites.google.com/site/PCADMIX. We use Singular Value Decomposition in R to perform PCA on the phased genotypes of the ancestral representatives. We project admixed individuals on the basis of principal components, and compute the observed ancestry “score” for a haplotype *i* in the *j* ^th^ window as the weighted average *L_j_g_ij_*, where *g_ij_* is a column vector of the haplotype's alleles (coded as 0 or 1) in window *j*, standardized by the mean and standard deviation of that SNP's frequency in the ancestral populations. *L_j_* is a matrix for which the entry in the *k* ^th^ row, *l* ^th^ column is the loading of SNP *l* in the window on principal component *k*. We use a forward-backward algorithm to identify the probability of ancestry at each window, conditional on the ancestry scores. For the forward-backward algorithm in our HMM, we used a haploid version of the transition and emission probabilities in the Viterbi algorithm of Bryc *et al.* [20]. The transition probability is defined by *p*, the probability of recombination between windows, and *q*_j_, the frequency of the target population's chromosomes in the admixing ancestral pool.

First, ancestral populations are thinned for SNPs with r^2^<0.8 in order to remove highly linked alleles from different populations, which can lead to spurious ancestry transitions. Second, chromosomes for each individual in a population are artificially strung together to create two haploid genomes for the individual; this step increases the amount of information used for PCA, and it is of special relevance given that Europeans, Near Easterners and North African are differentiated with an F_st_ of only ∼0.05. Then, PCA on a number *k*≤3 of ancestral populations is performed and the admixed population is projected into the determined *k*≤3 PCA space. PC loadings are used as weights in a weighted average of the allele values in a window of 40 SNPs. These window scores are then used as observed values in a HMM to assign posterior probabilities to the ancestry in each window (where chromosome were considered separately). Information on using PCADMIX in Egyptians is available in Figure S8. Additional performance testing and details of the implementation for this approach are available in [28], Texts S1, S2, S3 and Figure S9.

### Estimates of Migration Parameters

We tabulated the length and number of genomic tracts (i.e. phased haplotypes) assigned to particular population ancestries for the South Moroccan and Egyptian population samples (see above for PCA-based local ancestry assignment). We used a posterior probability threshold of 0.8, optimized for concordance with ADMIXTURE ancestry proportions (Figure 5A). The maximum likelihood estimate of the time of migration is sensitive to the minimum detectable length of migrant tracts. That is, as migrant tracts recombine with non-migrants and become smaller in size, we are less likely to detect them. Histograms of the cumulative number of migrant tracts of different lengths, for all individuals, were visualized (Figure S10) and we observe a reduction in the number of short migrant tracts in the 0.5 to 1.5 cM bins, inconsistent with constant or punctual migration model. Rather, this reduction can be understood as a reduction in our ability to detect short migrant segments due to insufficient SNP density or haplotype variation that is not present in our source population. We therefore choose a 3 cM threshold as the minimal length of migrant tracts to be considered. Theoretically, under an isolation followed by migration model and with a 3 cM tract length threshold, we have power to detect relatively recent migrations occurring within the past generations [30].

We modify Pool and Nielsen [30] equation 10, with 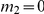 for the likelihood 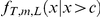 that a segment is of length 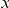 Morgans given that it is longer than the cutoff length 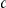 in a model with constant migration rate 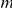 starting at time 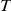 in a chromosome of length 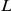. Similarly, we estimated a likelihood of 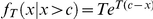 for punctuated migration occurring 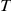 generations ago, which neglects chromosomal edge effects, an approximation justified by the fact that 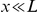 for a large majority of tracts.

## Supporting Information

Figure S1 A) *ADMIXTURE* results for k = 10 ancestral clusters in our North African populations, Spanish Basque, Near Eastern Qatari, western Africans, HapMap3 Kenyan Luhya and Maasai and Italian Tuscans. B) Log likelihoods for each of the *k* clusters tested.
(TIF)

Figure S2 We used multidimensional scaling (MDS) to discriminate clusters of genetic variation within Africa and neighboring regions. MDS was applied to the pairwise, individual identity-by-state (IBS) matrix of 279,500 SNPs using PLINK 1.07 software [45]. The component 3 versus 4 (A) and component 1 versus component 2 versus component 3 (B) were plotted together using R 2.11.1. Population colors match Figure S1A (*k* = 10). North African populations are all indicated in turquoise.
(TIF)

Figure S3 Long runs of homozygosity compared across North African populations and neighbors. –homozyg –homozyg-window-kb 5000 –homozyg-window-het 1 –homozyg-window-missing 1 –homozyg-snp 25 –homozyg-kb 500 –homozyg-gap 100.
(TIF)

Figure S4 Implementation of PCADMIX. A) A principal components analysis is first run for *k* = 3 ancestral populations. The proportion of Population A's ancestry in an admixed individual is estimated by: a given haplotype's (black square) distance from the line connecting the means of PCA1 and PC2 for the two other populations, as a proportion of the haplotype's distance from all edges. B) Simulated ancestry assignment with and without LD filtering. The black arrow indicates a region of simulated European ancestry that is incorrectly classified (at a posterior probability calling threshold of 0.9) as African when no linkage disequilbrium (LD) filtering is used, and whose ancestry is left undecided when LD filtering is implemented (r^2^<0.8).
(TIF)

Figure S5 Comparison of *ADMIXTURE* and *PCADMIX* ancestry estimations in (A) South Moroccans and (B) Egyptians. In both cases *PCADMIX* was required to assign ancestry with a posterior probability of 0.95. The 0.95 threshold substantially reduces the proportion of the genome assigned by PCADMIX. In South Moroccans, the reduction in assigned ancestry occurs primarily in the European and to a lesser extent in the Berber component. For the Egyptians, the reduction in assigned ancestry is dramatically reduce Near Eastern (or Arabic) ancestry.
(TIF)

Figure S6 A) PCADMIX applied to a South Moroccan individual using Saharawi, Basques and Luhyan as ancestral populations. Segments are assigned to ancestries with a posterior probability higher than 0.8. B) PCADMIX applied to the same South Moroccan individual as in A) using Tunisian, Basque and Luhya as the ancestral populations. Segments are assigned to ancestries with a posterior probability higher than 0.8.
(TIF)

Figure S7 We capture admixture proportions by independently running LAMP [29] for estimating local ancestry using the Tunisian Berber, European Basque and sub-Saharan Luhya source populations. Sub-Saharan ancestry appears concordant with ADMIXTURE and PCADMIX. Tracts of “Maghrebi” ancestry appear shorter than those inferred in PCADMIX, although this may be attributed to the use of the high Maghrebi but low diversity Tunisian Berbers. Results are shown for chromosome 1 (A) and X chromosome (B).
(TIF)

Figure S8 Shown is the admixture deconvolution for chromosome 1 using PCADMIX for 19 Egyptian individuals (*n* = 38). Initially we assigned ancestry for *k* = 3 ancestral populations (Maghreb: SAH, European: BAS, Sub-Saharan: MKK) using a 0.8 posterior probability threshold, shown in (A,B). Then we assumed a different set of 3 ancestral populations (Maghreb: SAH, European: BAS, Near Eastern: QAT) shown in (C,D). In the third step, we assumed the Sub-Saharan ancestry, assigned in A, represented truly divergent sub-Saharan haplotypes given the high F_st_ between this ancestry and all others. E) We layered these haplotypes on top of [C] (Maghreb, European, Near Eastern) deconvoluted chromosomes.
(TIF)

Figure S9 A) We present the average assigned ancestry (>0.8 posterior probability) across chromosome 1 for each of 4 ancestries assigned in the Egyptians: Maghrebi (Saharawi), European (Basque), Near Eastern (Qatari), Sub-Saharan (Maasai).
(TIF)

Figure S10 A) Distribution of the number and length in centimorgans of migrant Sub-Saharan (Luhya) tracts distributed by length found in the South Moroccan population. B) Distribution of the number and length in centimorgans of migrant Sub-Saharan (Maasai) tracts distributed by length found in the Egyptian population. Red bar indicates the minimum threshold cutoff employed in the migration parameter analysis. Please note the different scales along the X-axis.
(TIF)

Table S1 Name, sample size and country of origin for populations newly genotyped in the present study as well as for populations published previously. References are included in the table.
(DOC)

Table S2 Additional estimates of F_st_ after removed putative admixture events.
(DOC)

Table S3 Significance of the comparisons of ancestry assignment using PCADMIX and *ADMIXTURE*.
(DOC)

Text S1 Assigning local ancestry with PCADMIX.
(DOC)

Text S2 Concordance between ADMIXTURE and PCADMIX.
(DOC)

Text S3 Chromosome 1 Ancestry Deviations.
(DOC)
