# Supplementary figures and images for: Genomic Ancestry of North Africans Supports Back-to-Africa Migrations
Source: PLoS Genet. 2012 Jan 12;8(1):e1002397. doi: 10.1371/journal.pgen.1002397 (PMC3257290; doi:10.1371/journal.pgen.1002397)

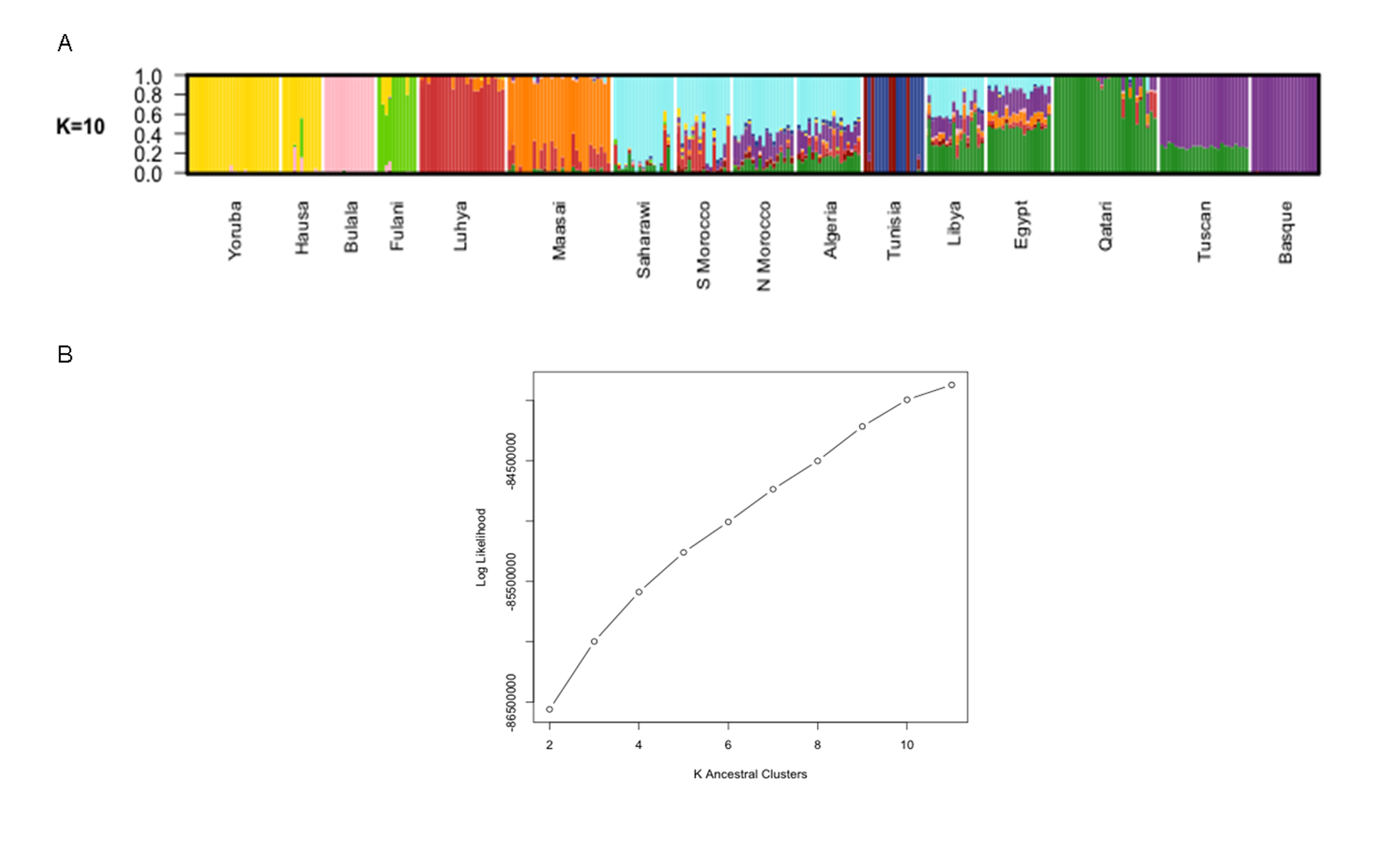

Supplement: Figure S1 — A) ADMIXTURE results for k = 10 ancestral clusters in our North African populations, Spanish Basque, Near Eastern Qatari, western Africans, HapMap3 Kenyan Luhya and Maasai and Italian Tuscans. B) Log likelihoods for each of the k clusters tested. (TIF) [file pgen.1002397.s001.tif]

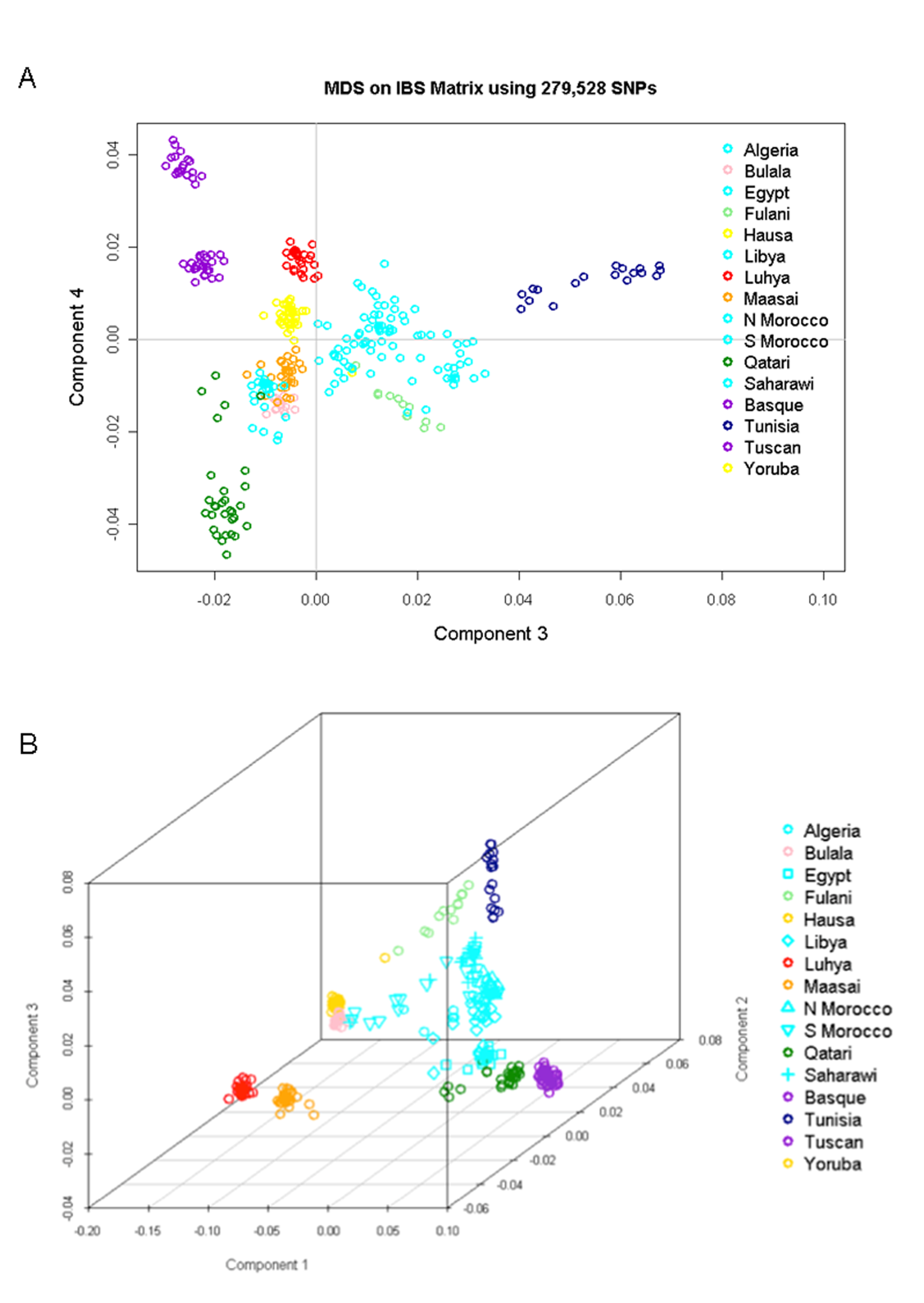

Supplement: Figure S2 — We used multidimensional scaling (MDS) to discriminate clusters of genetic variation within Africa and neighboring regions. MDS was applied to the pairwise, individual identity-by-state (IBS) matrix of 279,500 SNPs using PLINK 1.07 software [45]. The component 3 versus 4 (A) and component 1 versus component 2 versus component 3 (B) were plotted together using R 2.11.1. Population colors match Figure S1A (k = 10). North African populations are all indicated in turquoise. (TIF) [file pgen.1002397.s002.tif]

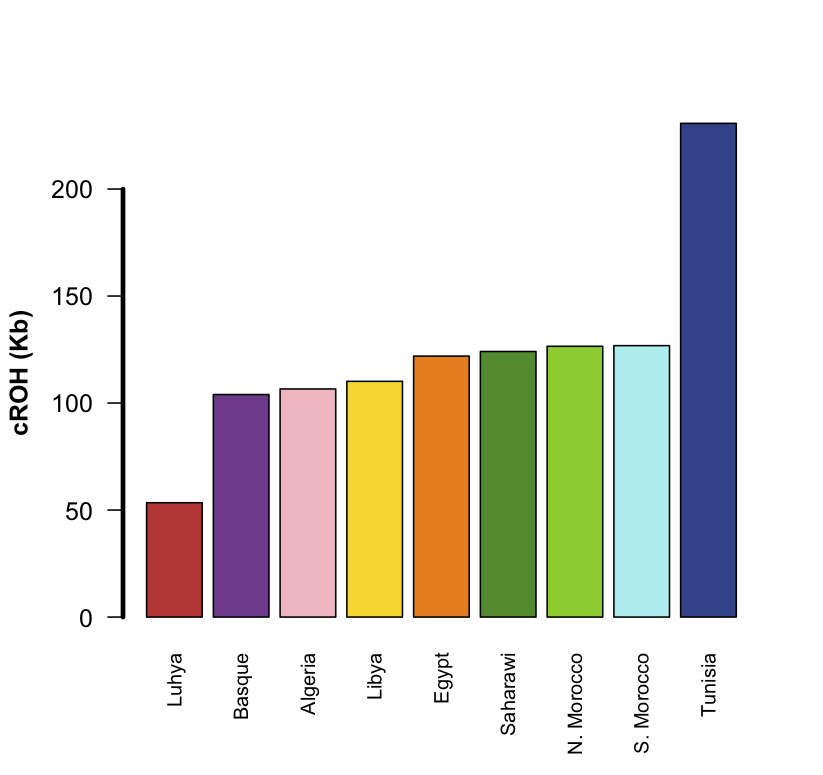

Supplement: Figure S3 — Long runs of homozygosity compared across North African populations and neighbors. –homozyg –homozyg-window-kb 5000 –homozyg-window-het 1 –homozyg-window-missing 1 –homozyg-snp 25 –homozyg-kb 500 –homozyg-gap 100. (TIF) [file pgen.1002397.s003.tif]

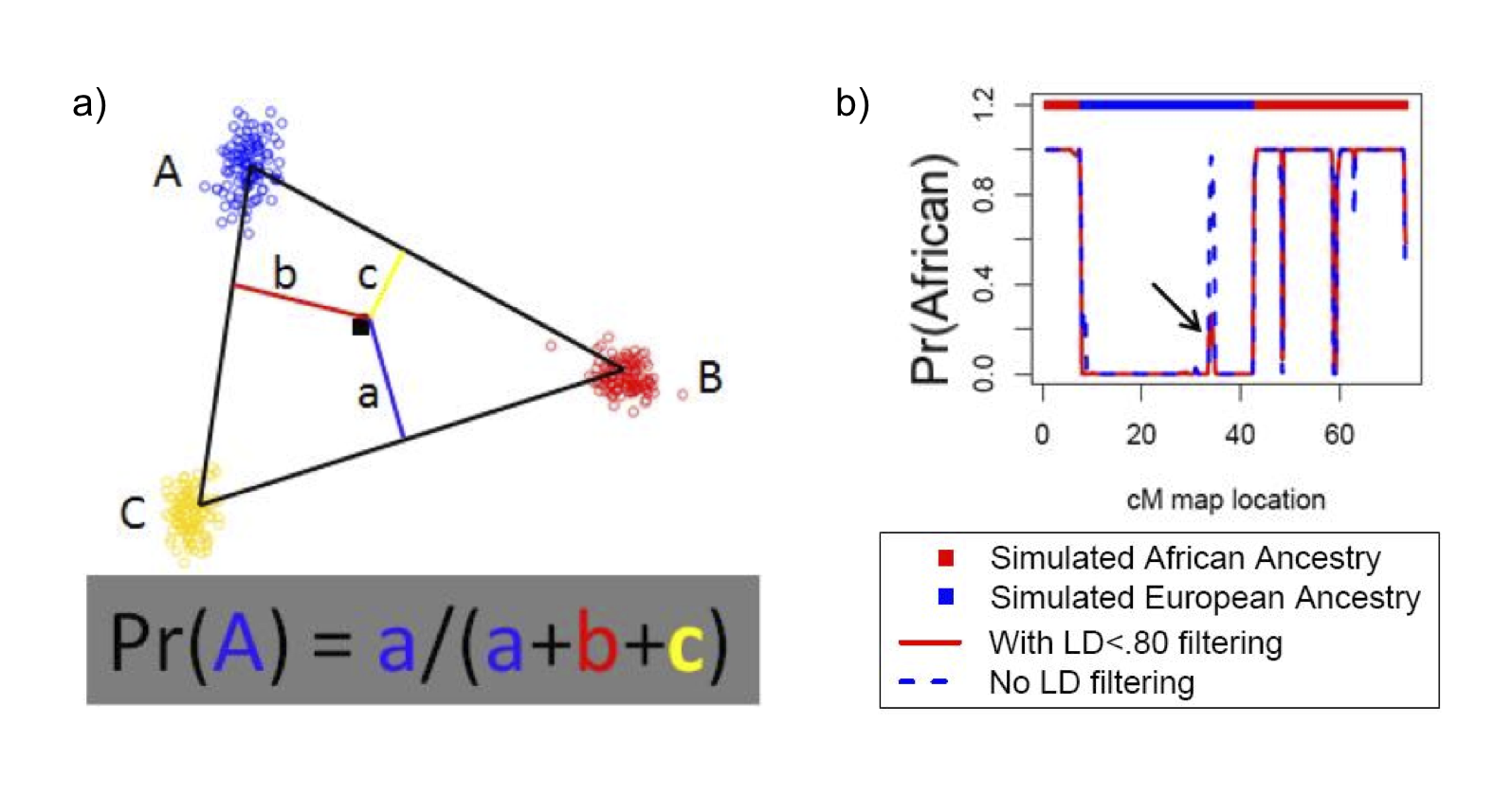

Supplement: Figure S4 — Implementation of PCADMIX. A) A principal components analysis is first run for k = 3 ancestral populations. The proportion of Population A's ancestry in an admixed individual is estimated by: a given haplotype's (black square) distance from the line connecting the means of PCA1 and PC2 for the two other populations, as a proportion of the haplotype's distance from all edges. B) Simulated ancestry assignment with and without LD filtering. The black arrow indicates a region of simulated European ancestry that is incorrectly classified (at a posterior probability calling threshold of 0.9) as African when no linkage disequilbrium (LD) filtering is used, and whose ancestry is left undecided when LD filtering is implemented (r2<0.8). (TIF) [file pgen.1002397.s004.tif]

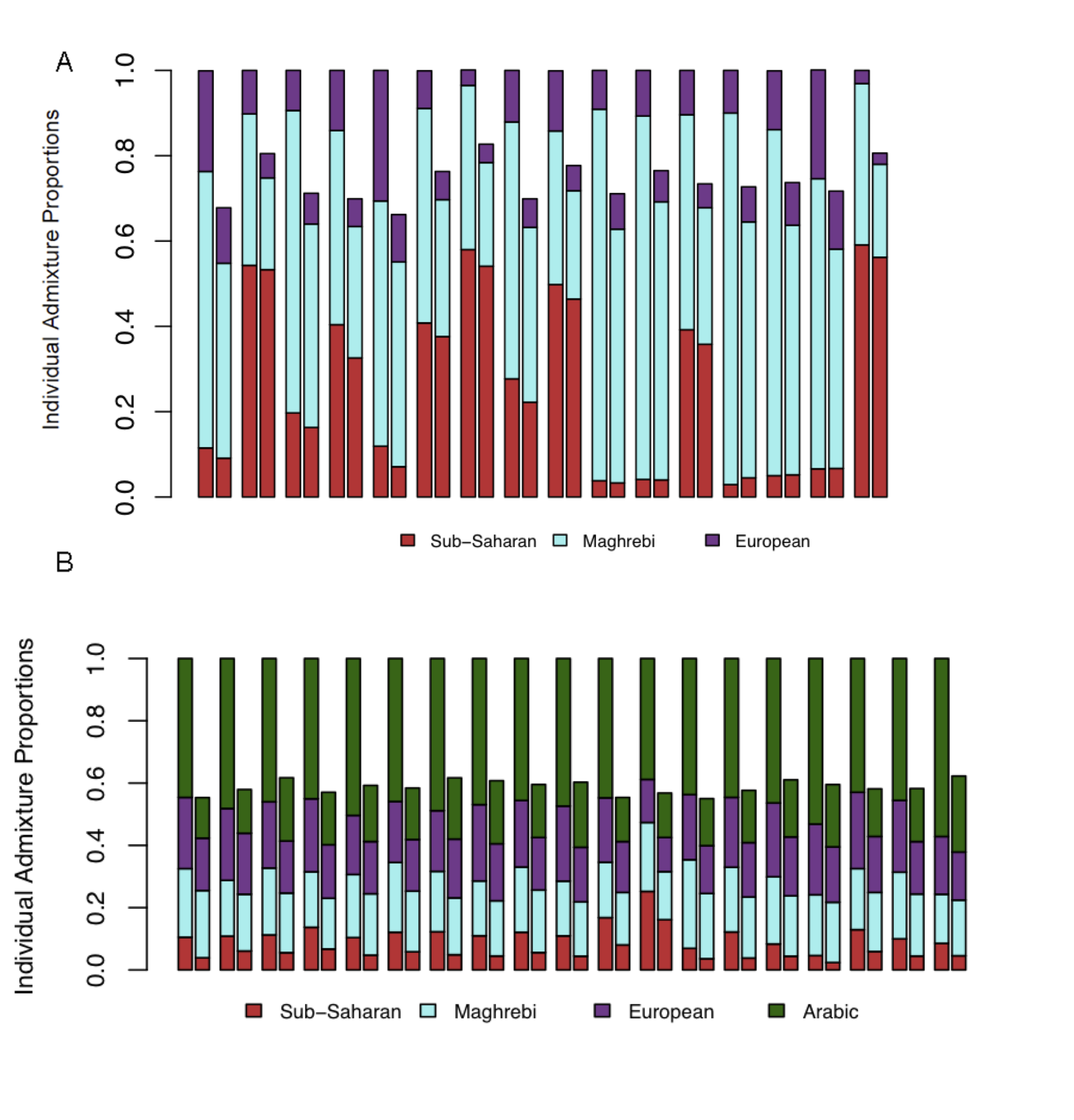

Supplement: Figure S5 — Comparison of ADMIXTURE and PCADMIX ancestry estimations in (A) South Moroccans and (B) Egyptians. In both cases PCADMIX was required to assign ancestry with a posterior probability of 0.95. The 0.95 threshold substantially reduces the proportion of the genome assigned by PCADMIX. In South Moroccans, the reduction in assigned ancestry occurs primarily in the European and to a lesser extent in the Berber component. For the Egyptians, the reduction in assigned ancestry is dramatically reduce Near Eastern (or Arabic) ancestry. (TIF) [file pgen.1002397.s005.tif]

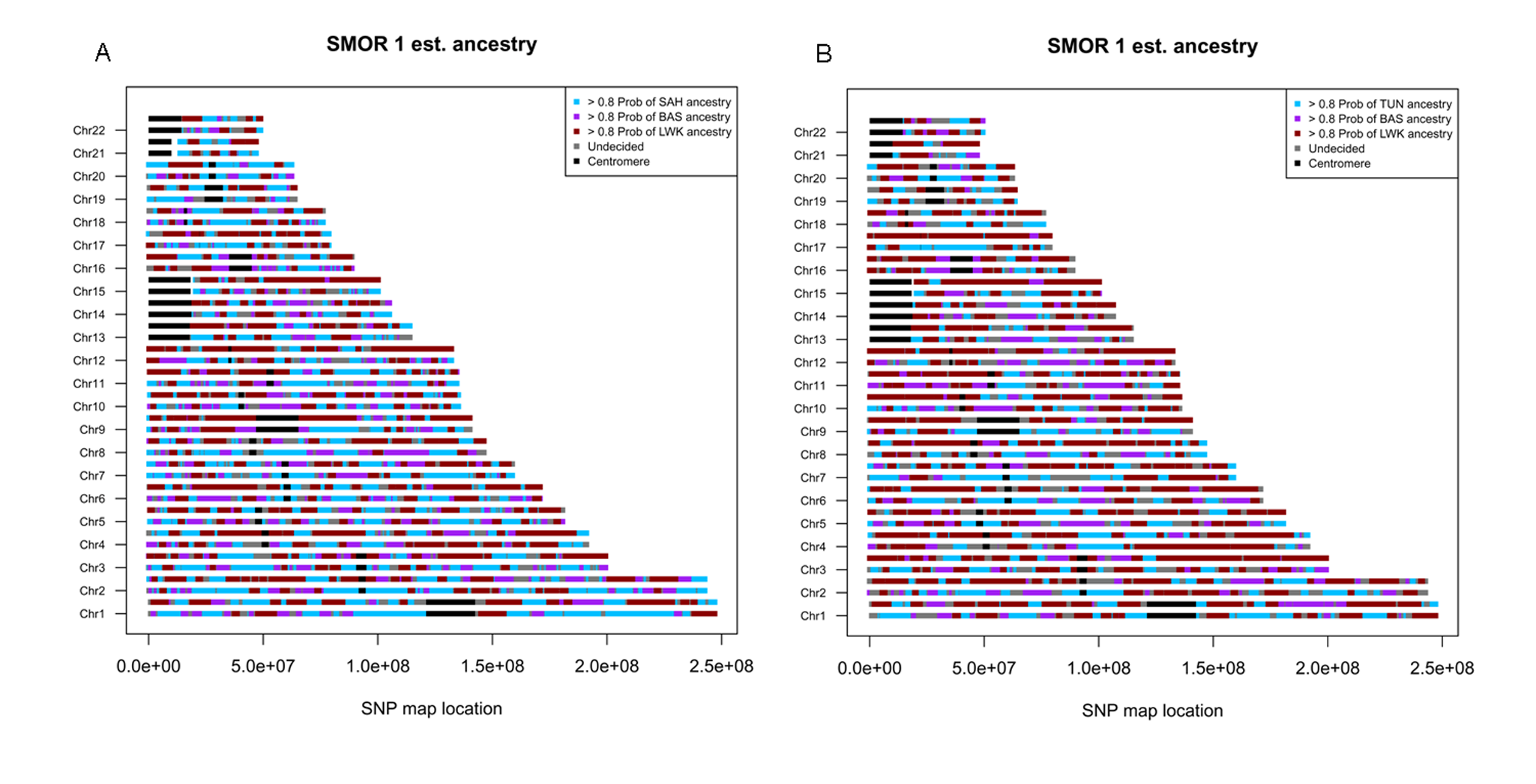

Supplement: Figure S6 — A) PCADMIX applied to a South Moroccan individual using Saharawi, Basques and Luhyan as ancestral populations. Segments are assigned to ancestries with a posterior probability higher than 0.8. B) PCADMIX applied to the same South Moroccan individual as in A) using Tunisian, Basque and Luhya as the ancestral populations. Segments are assigned to ancestries with a posterior probability higher than 0.8. (TIF) [file pgen.1002397.s006.tif]

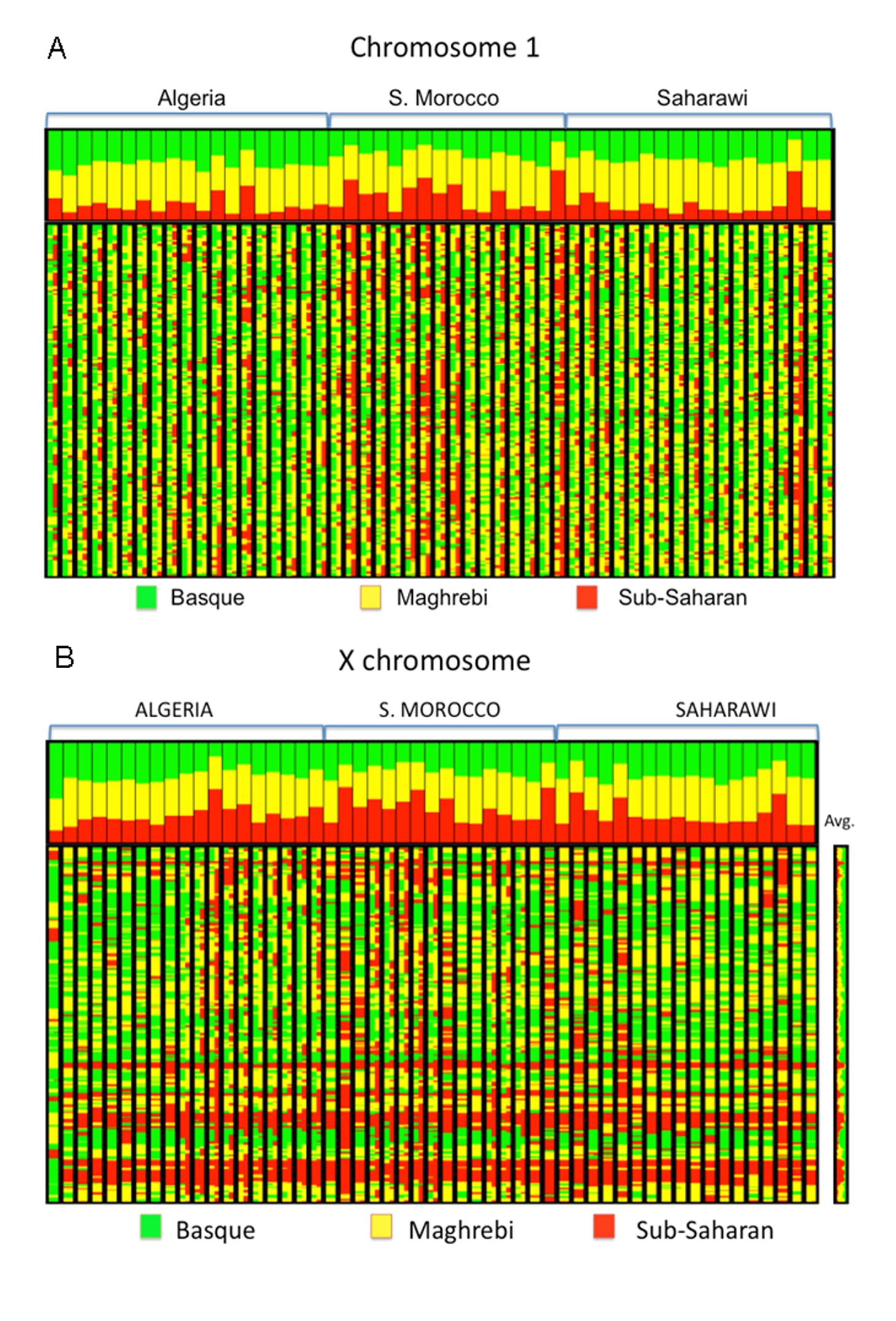

Supplement: Figure S7 — We capture admixture proportions by independently running LAMP [29] for estimating local ancestry using the Tunisian Berber, European Basque and sub-Saharan Luhya source populations. Sub-Saharan ancestry appears concordant with ADMIXTURE and PCADMIX. Tracts of “Maghrebi” ancestry appear shorter than those inferred in PCADMIX, although this may be attributed to the use of the high Maghrebi but low diversity Tunisian Berbers. Results are shown for chromosome 1 (A) and X chromosome (B). (TIF) [file pgen.1002397.s007.tif]

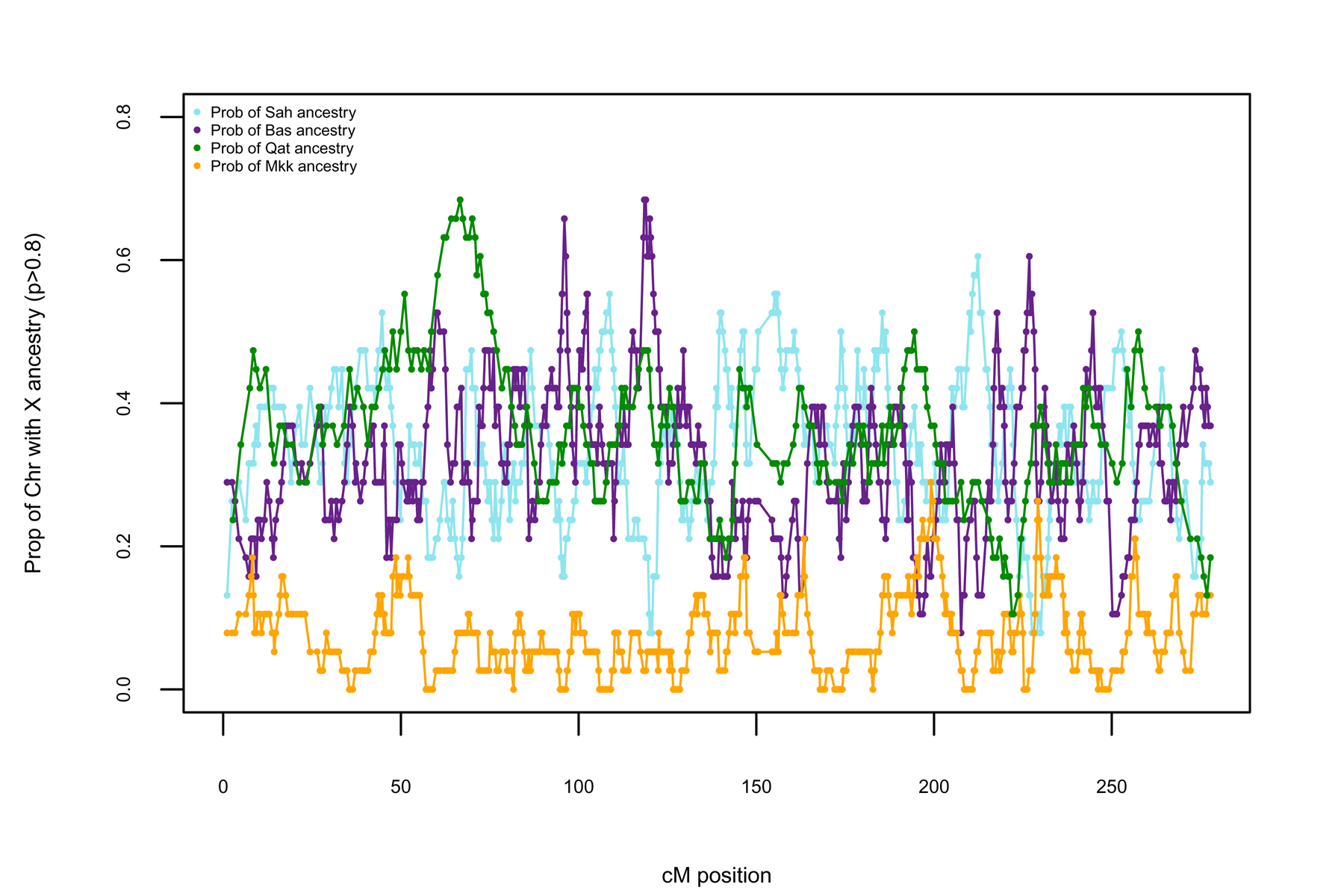

Supplement: Figure S8 — Shown is the admixture deconvolution for chromosome 1 using PCADMIX for 19 Egyptian individuals (n = 38). Initially we assigned ancestry for k = 3 ancestral populations (Maghreb: SAH, European: BAS, Sub-Saharan: MKK) using a 0.8 posterior probability threshold, shown in (A,B). Then we assumed a different set of 3 ancestral populations (Maghreb: SAH, European: BAS, Near Eastern: QAT) shown in (C,D). In the third step, we assumed the Sub-Saharan ancestry, assigned in A, represented truly divergent sub-Saharan haplotypes given the high Fst between this ancestry and all others. E) We layered these haplotypes on top of [C] (Maghreb, European, Near Eastern) deconvoluted chromosomes. (TIF) [file pgen.1002397.s008.tif]

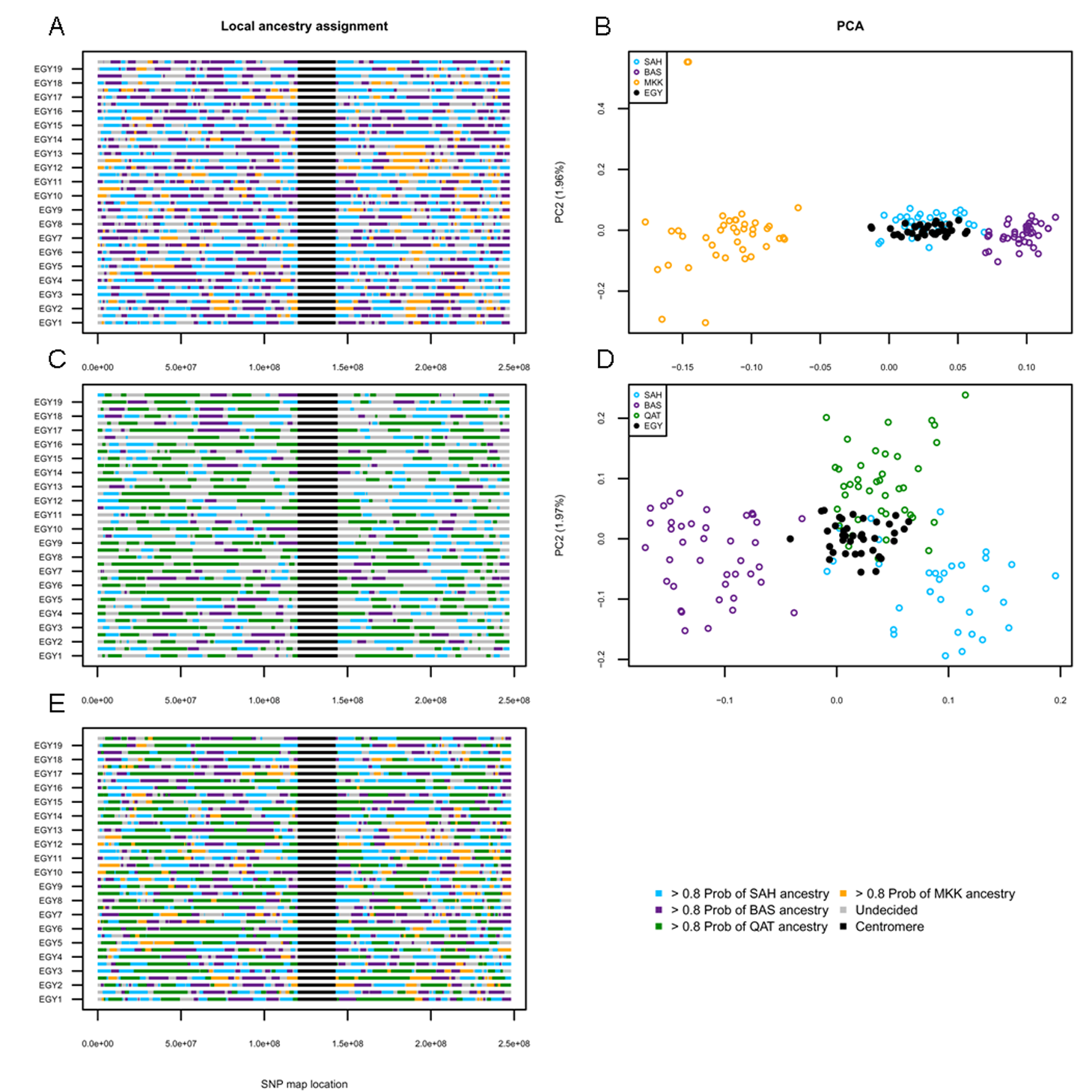

Supplement: Figure S9 — A) We present the average assigned ancestry (>0.8 posterior probability) across chromosome 1 for each of 4 ancestries assigned in the Egyptians: Maghrebi (Saharawi), European (Basque), Near Eastern (Qatari), Sub-Saharan (Maasai). (TIF) [file pgen.1002397.s009.tif]

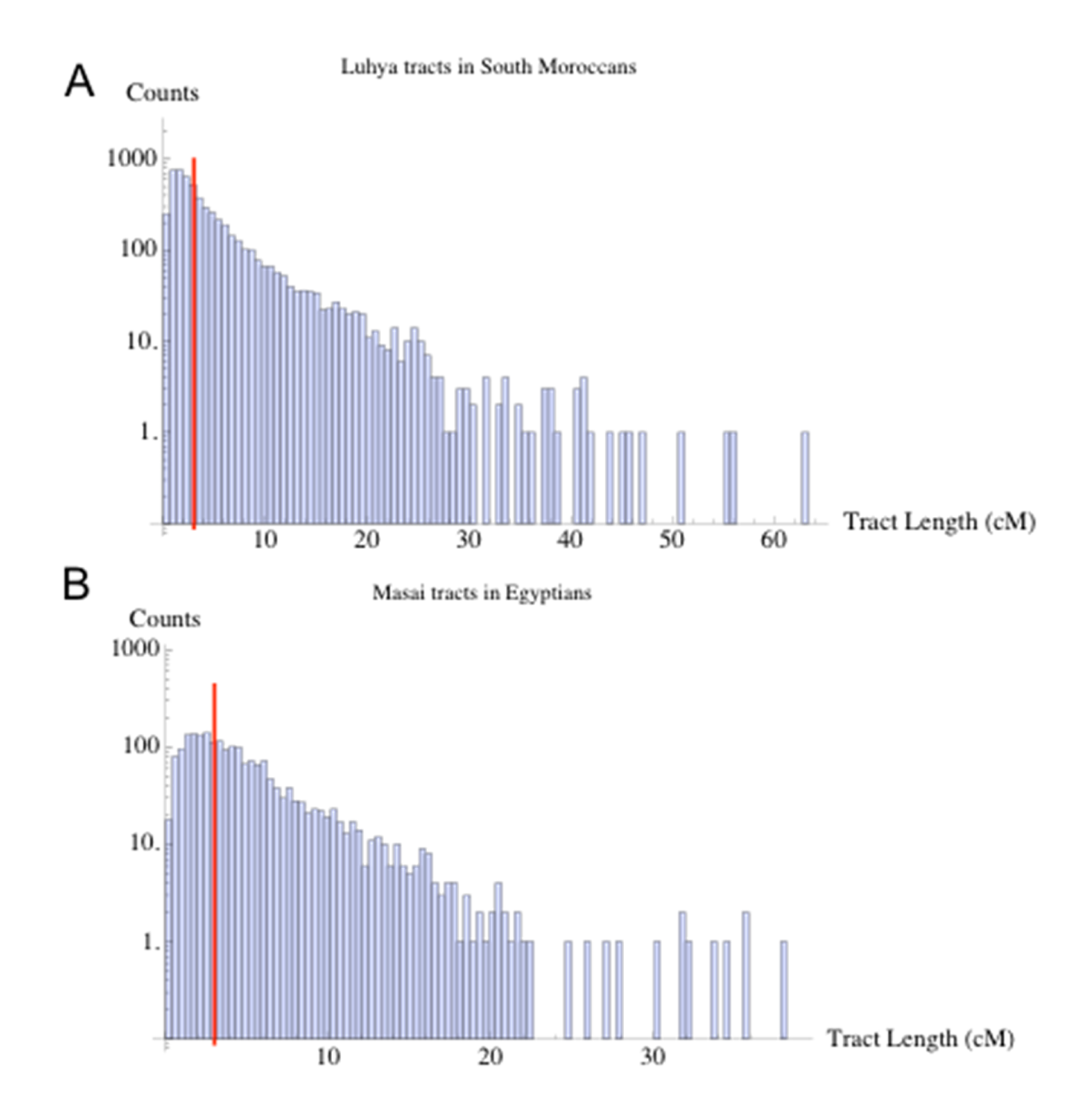

Supplement: Figure S10 — A) Distribution of the number and length in centimorgans of migrant Sub-Saharan (Luhya) tracts distributed by length found in the South Moroccan population. B) Distribution of the number and length in centimorgans of migrant Sub-Saharan (Maasai) tracts distributed by length found in the Egyptian population. Red bar indicates the minimum threshold cutoff employed in the migration parameter analysis. Please note the different scales along the X-axis. (TIF) [file pgen.1002397.s010.tif]
