# Supplementary material for: Genomic Ancestry of North Africans Supports Back-to-Africa Migrations
Source: PLoS Genet. 2012 Jan 12;8(1):e1002397. doi: 10.1371/journal.pgen.1002397 (PMC3257290; doi:10.1371/journal.pgen.1002397)
Supplement: Table S1 — Name, sample size and country of origin for populations newly genotyped in the present study as well as for populations published previously. References are included in the table. (DOC) [file pgen.1002397.s011.doc]

**Table S1:**

Details of the dataset used in the present study.

| **Population** | **Sample Size** | **Country** | **Reference** |
| --- | --- | --- | --- |
| Morocco - North | 18 | Morocco | *Present study* |
| Morocco - South | 16 | Morocco | *Present study* |
| Saharawi | 18 | Western Sahara | *Present study* |
| Algerian | 19 | Algeria | *Present study* |
| Tunisian | 18 | Tunisia | *Present study* |
| Libyan | 17 | Libya | *Present study* |
| Egyptian | 19 | Egypt | *Present study* |
| Basques | 20 | Spain | *Present study* |
| Tuscans | 26 | Italy | HapMap3 |
| Qatari | 30 | Qatar | Hunter-Zinck et al. 2010 |
| Yoruba | 26 | Nigeria | HapMap3 |
| Hausa | 12 | Nigeria | Bryc et al. 2010 |
| Bulala | 15 | Nigeria | Bryc et al. 2010 |
| Fulani | 12 | Nigeria | Bryc et al. 2010 |
| Luhya | 25 | Kenya | HapMap3 |
| Maasai | 30 | Kenya | HapMap3 |
