# Supplementary material for: Genomic Ancestry of North Africans Supports Back-to-Africa Migrations
Source: PLoS Genet. 2012 Jan 12;8(1):e1002397. doi: 10.1371/journal.pgen.1002397 (PMC3257290; doi:10.1371/journal.pgen.1002397)
Supplement: Table S2 — Additional estimates of Fst after removed putative admixture events. (DOC) [file pgen.1002397.s012.doc]

**Table S2:**

*One tailed t-tests comparing ancestry assigned in PCADMIX and ADMIXTURE*

| **Admixed Population** | **Assigned Ancestry** | **p-value Posterior Prob. >0.8** | **p-value Posterior Prob. >0.95** |
| --- | --- | --- | --- |
| South Moroccan | Luhya (East African) | 0.845 | 0.734 |
| South Moroccan | Maghreb (Saharawi) | 0.285 | 0.005** |
| South Moroccan | European (Basque) | 0.988 | 0.015* |
| Egyptian | Maasai (East African) | <0.01** | - |
| Egyptian | Maghreb (Saharawi) | <0.01** | - |
| Egyptian | European (Basque) | <0.01** | - |
| Egyptian | Near Eastern (Qatari) | <0.01** | - |

* Indicates significance assessed with p <0.05 and ** indicates significance at p <0.01.
