# Supplementary material for: Genomic Ancestry of North Africans Supports Back-to-Africa Migrations
Source: PLoS Genet. 2012 Jan 12;8(1):e1002397. doi: 10.1371/journal.pgen.1002397 (PMC3257290; doi:10.1371/journal.pgen.1002397)
Supplement: Table S3 — Significance of the comparisons of ancestry assignment using PCADMIX and ADMIXTURE. (DOC) [file pgen.1002397.s013.doc]

**Table S3**

*F_st_ estimates for K=8 using ADMIXTURE*

| **K=8** | **Masaai** | **Qatari** | **Maghrebi** | **W Africa** | **Luhya** | **Tunisia** | **Bulala** |
| --- | --- | --- | --- | --- | --- | --- | --- |
| **Qatari** | 0.117 |  |  |  |  |  |  |
| **Maghrebi** | 0.093 | 0.055 |  |  |  |  |  |
| **W Africa** | 0.083 | 0.193 | 0.158 |  |  |  |  |
| **Luhya** | 0.052 | 0.165 | 0.133 | 0.062 |  |  |  |
| **Tunisia** | 0.126 | 0.074 | 0.07 | 0.194 | 0.166 |  |  |
| **Bulala** | 0.076 | 0.185 | 0.15 | 0.045 | 0.072 | 0.187 |  |
| **European** | 0.137 | 0.05 | 0.059 | 0.211 | 0.184 | 0.079 | 0.203 |
