# Supplementary material for: Genomic Ancestry of North Africans Supports Back-to-Africa Migrations
Source: PLoS Genet. 2012 Jan 12;8(1):e1002397. doi: 10.1371/journal.pgen.1002397 (PMC3257290; doi:10.1371/journal.pgen.1002397)
Supplement: Text S1 — Assigning local ancestry with PCADMIX. (DOC) [file pgen.1002397.s014.doc]

**Text S1. Assigning local ancestry with PCADMIX**

PCADMIX accurately distinguishes ancestries that are highly genetically diverged, for instance differentiating Native American or European from African ancestry (Brisbin 2010). We implemented PCADMIX for South Moroccans and Egyptians, where the choice of k=3 ancestral populations was informed by ADMIXTURE results (Figure 1). Moroccans have four primary ancestries: sub-Saharan, Maghrebi, European and Near Eastern. The Maghrebi, European and Near Eastern populations tend to be less genetically diverged than typical sources populations used for local ancestry deconvolution (e.g. European and west African for African-Americans). The Moroccan sub-Saharan component appears closest to the Bantu-speaking Luhya (LWK) population; the West Saharan Saharawi represented the Maghreb component (two Saharawi individuals with high sub-Saharan ancestry were removed); and the Spanish Basque (BAS) carried the highest amount of European component and it was therefore selected as the representative of European ancestry. A small percent of Near Eastern ancestry <5% was identified in the ADMIXTURE analysis (Figure 1), but was not incorporated into the PCADMIX analysis. Egyptians had appreciable ancestry from four similar populations, but the representative of sub-Saharan ancestry based on ADMIXTURE *k*=6:8 appeared to be closest to the Kenyan Maasai (MKK) rather than Luhya.

We first ran *PCADMIX* separately for each chromosome (1:22) for the admixed South Moroccan population. We found that in the principal component analysis, PC2 did not separate the assumed ancestral populations for some chromosomes, probably due to the lower genetic differentiation existing among the non-African populations compared to that between them and the sub-Saharan population. In order to increase the power of *PCADMIX* to separate the ancestral populations in the PCA step we aimed to increase the amount of information available for discrimination, in this case the number of SNPs. We thus strung together all the chromosomes to create the initial PC1 vs. PC2 instead of running the PCA chromosome by chromosome. With this modification PC2 clearly separated the ancestral populations and tended to cluster the individuals that belonged to the same population. After obtaining the PC loading for 40 SNP haplotypes, the HMM is run on a chromosome by chromosome basis. From PCA and ADMIXTURE analysis, we also detected two Western Sahara individuals as outliers from the Maghrebi ancestral population; these individual had higher components of sub-Saharan ancestry. Given that our aim was to use the Western Saharawi as an ancestral population for the Maghrebi component, those 2 individuals were removed from the analysis.

Population genotype data were thinned for linkage (r^2^ <0.8) prior to PCA. SNPs in very high linkage disequilbrium can provide additional support for a particular ancestry in a given window. Highly linked SNPs contribute very similar information about population membership but inferred population membership for very small windows may be spurious. The combined evidence from multiple SNPs in high LD can potentially outweigh the evidence from a smaller number of nearby but less linked SNPs, resulting in spurious ancestry transitions, as demonstrated in Figure S4. We believe an LD filtering step improves the quality of long haplotype assignment.
