# Supplementary material for: Genomic Ancestry of North Africans Supports Back-to-Africa Migrations
Source: PLoS Genet. 2012 Jan 12;8(1):e1002397. doi: 10.1371/journal.pgen.1002397 (PMC3257290; doi:10.1371/journal.pgen.1002397)
Supplement: Text S2 — Concordance between ADMIXTURE and PCADMIX. (DOC) [file pgen.1002397.s015.doc]

**Text S2. Concordance between ADMIXTURE and PCADMIX**

Assuming that individuals’ proportions of each ancestry are normally distributed within the population, we used a two-tailed *t-test* to ask if there was a statistical difference between ancestry proportions estimated with ADMIXTURE versus PCADMIX (Table S2). When we required ancestry tracts in PCADMIX to have >0.80 posterior probability, there was no significant difference in ancestry proportions in the two different methods for South Moroccans. When we required ancestry tracts to exceed a >0.95 posterior probability threshold, the Maghrebi and European ancestry proportions differed significantly from ADMIXTURE estimates (Figure S5A, Table S2); both of these ancestries were underestimated in our PCADMIX model. For this set of populations we considered 0.95 to be too stringent, and further analyses were conducted with the 0.80 threshold. Ancestry proportions across individuals were highly correlated between the two methods, suggesting that even if particular ancestries tended to be underestimated, this was true for most individuals.

We additionally considered the source populations used for possible admixture deconvolution in the Egyptian population. With a posterior probability threshold >0.8, the three Mediterranean ancestries [Maghrebi, European, Near Eastern] significantly differed from the corresponding population estimates from ADMIXTURE (Table S2). Interestingly for Egyptians, PCADMIX typically assigned excessive amounts of Maghrebi ancestry (difference in means was 10%), while apparently underestimating Near Eastern ancestry by 11%. The European ancestry differed by only 3% (Figure S5B).
