# Supplementary material for: Genomic Ancestry of North Africans Supports Back-to-Africa Migrations
Source: PLoS Genet. 2012 Jan 12;8(1):e1002397. doi: 10.1371/journal.pgen.1002397 (PMC3257290; doi:10.1371/journal.pgen.1002397)
Supplement: Text S3 — Chromosome 1 Ancestry Deviations. (DOC) [file pgen.1002397.s016.doc]

**Text S3. Chromosome 1 Ancestry Deviations**

For the southern Moroccan and Egyptian populations we plot deviations from the average assigned ancestry along chromosome 1, where the posterior probability for an assigned ancestry was required to be at least 0.80 (Figure 5A, Figure S8). In 40 SNP windows we show the difference from the locus-specific and average ancestry. In Egyptians, deviation from the average ancestry was generally on the order of ±22%. The over- and under-representation of Near Eastern Qatari ancestry is especially pronounced around 65 cM (Figure S9). The assigned Maghrebi and Basque ancestries appear to have an inverse relationship, even though the two populations form disparate clusters in PCA spaces (Figure S8). For large population samples, we expect substantial deviations from the average ancestry at a locus to be due to: natural selection, spurious ancestry spikes due to linkage disequilibrium or population structure between the source and admixed populations. However, our North African sample sizes are small (*n*<40) and sampling variance alone may explain the fluctuations in average ancestry.
